# Association Between Social Isolation and Smoking in Japan and England

**DOI:** 10.2188/jea.JE20200138

**Published:** 2021-10-05

**Authors:** Takaaki Ikeda, Noriko Cable, Masashige Saito, Shihoko Koyama, Taishi Tsuji, Taiji Noguchi, Katsunori Kondo, Ken Osaka, Jun Aida

**Affiliations:** 1 Department of Health Policy Science, Graduate School of Medical Science, Yamagata University, Yamagata, Japan; 2 Department of International and Community Oral Health, Tohoku University Graduate School of Dentistry, Sendai, Japan; 3 Department of Epidemiology and Public Health, University College London, London, United Kingdom; 4 Department of Social Welfare, Nihon Fukushi University, Aichi, Japan; 5 Cancer Control Center, Osaka International Cancer Institute, Osaka, Japan; 6 Faculty of Health and Sport Sciences, University of Tsukuba, Tokyo, Japan; 7 Department of Social Science, National Center for Geriatrics and Gerontology, Aichi, Japan; 8 Department of Social Preventive Medical Sciences, Center for Preventive Medical Sciences, Chiba University, Chiba, Japan; 9 Department of Oral Health Promotion, Graduate School of Medical and Dental Sciences, Tokyo Medical and Dental University, Tokyo, Japan; 10 Division for Regional Community Development, Liaison Center for Innovative Dentistry, Graduate School of Dentistry, Tohoku University, Sendai, Japan

**Keywords:** repeated cross-sectional study, ELSA, JAGES, social isolation, smoking status

## Abstract

**Background:**

Existing evidence suggest that those who are socially isolated are at risk for taking up or continuing smoking. This study investigated country-based differences in social isolation and smoking status.

**Methods:**

We performed a repeated cross-sectional study using two waves of data from two ongoing aging studies: the English Longitudinal Study of Ageing and the Japan Gerontological Evaluation Study. Participants from both studies aged ≥65 years were included. We applied a multilevel Poisson regression model to examine the association between social isolation and smoking status and adjusted for individual sociodemographic characteristics. We used the social isolation index which comprises the following domains: marital status; frequency of contact with friends, family, and children; and participation in social activities. Interaction terms between each country and social isolation were also entered into the mode.

**Results:**

After exclusion of never smokers, we analyzed 75,905 participants (7,092 for ELSA and 68,813 for JAGES, respectively). Taking ex-smokers as the reference, social isolation was significantly associated with current smoking; the prevalence ratios (PRs) were 1.06 (95% credible interval [CrI], 1.05–1.08) for men and 1.08 (95% CrI, 1.04–1.11) for women. Taking Japan as a reference, the interaction term between country and social isolation was significant for both sexes, with increased PRs of 1.32 (95% CrI, 1.14–1.50) for men and 1.30 (95% CrI, 1.11–1.49) for women in England.

**Conclusions:**

Older people who were less socially isolated were more likely to quit smoking in England than in Japan, possibly explained by the strict tobacco control policies in England.

## INTRODUCTION

Smoking continues to be one of the leading global causes of cardiovascular-related diseases and mortality.^1^^,^^2^ The prevalence of smoking is high worldwide, especially among men, and in 2015, 35% of men and 6% of women were reported as smokers.^2^ Once people start to smoke, the addiction to tobacco smoking remains, even in those who quit smoking.^3^ Thus, tobacco control is an important public health issue.

A cross-sectional study from South Korea showed that more extensive social networks, such as having a partner, friends, relatives, and social activities, were negatively associated with current smoking status among older women.^4^ In a cohort study conducted in the United States,^5^ social network effects of smoking cessation were also supported by a significant and positive association between smoking cessation by family members, relatives, and friends and smoking cessation of the study participants. In contrast, social isolation, defined as a state in which objectively quantifiable social interactions, contacts, and networks are absent,^6^^–^^8^ was associated with smoking, meaning that socially isolated individuals are likely to be smokers.^6^

The World Health Organization supports the upward implementation to impose high tobacco taxes, driving up retail prices of the product, as the most effective tobacco control measure.^2^^,^^9^^,^^10^ Higher retail prices have been found to reduce the prevalence of smoking in the older population at a rate of approximately 9% for every 1 United States dollar (USD) increase.^10^ However, the retail prices of tobacco across countries, especially those in Asia, have remained low. For example, a pack of 20 cigarettes in Japan costs 4.18 USD, which is far cheaper compared with the prices in European countries, especially in England, where the difference is >8 USD.^11^

Smoking prevalence tends to be high in countries where tobacco control policies are more lenient, given that smoking is considered a macro-level norm.^2^^,^^9^^,^^10^ Social network effects on smoking cessation in Asian countries^4^^,^^12^ and the United States^5^ have been reported as varied, which could be because these effects are likely to be suppressed in countries with a high prevalence of smoking and weak tobacco control policies, such as low tobacco taxes. However, little is known regarding whether the association between social isolation and smoking differs between ex-smokers and current smokers because ex-smokers are at higher risk for smoking relapse than those who have never smoked.^3^ Furthermore, the association between social networks and smoking cessation has not been fully explored in cross-national comparative studies. Therefore, we conducted this study to elucidate differences in the association of social isolation on smoking status between Japan and England using large-scale data.

## METHODS

### Study population

Our repeated cross-sectional study utilized the data from two ongoing aging studies: the English Longitudinal Study of Ageing (ELSA) and the Japan Gerontological Evaluation Study (JAGES). The ELSA is a nationally representative survey, while the JAGES is not a representative one that is conducted in the collaborating municipalities. However, the municipalities were located nationwide, from 16 out of 47 prefectures, and the participants in each municipality were selected by representative sampling. The ELSA survey has been conducted every 2 years, while the JAGES survey has been conducted every 3 years. We used two waves of data where the survey years corresponded closely (2010–2011 and 2012–2013 for ELSA and 2010–2012 and 2013 for JAGES). Detailed descriptions of these studies have been provided elsewhere.^6^^,^^7^^,^^13^^,^^14^

The ELSA includes independent-living participants in England aged ≥50 years old, whereas independent-living adults aged ≥65 years old were targeted in the JAGES. To make the results comparable, we used the respondents aged ≥65 old in two waves of the ELSA data, consisting of an analytical sample of 5,068 men (2010–2011, *n* = 2,449; 2012–2013, *n* = 2,619) and 5,994 women (2010–2011, *n* = 2,928; 2012–2013, *n* = 3,066) for the ELSA and 107,411 men (2010–2012, *n* = 47,289; 2013, *n* = 60,122) and 125,198 women (2010–2012, *n* = 55,580; 2013, *n* = 69,618) for the JAGES.

### Measurements

As the outcome, participants’ self-reported current smoking status was categorized as current smoker, ex-smoker, and never smoker. For the ELSA survey, participants were asked if they had ever smoked. Those participants who responded “yes” were further asked whether or not they smoked at present. Participants who responded “yes” to the first question and “no” to the second one were classified as ex-smokers. For the JAGES survey, participants were asked their smoking status and were classified as ex-smokers if they responded “I used to smoke.”

For the explanatory factor, we applied a composite measure of social isolation, as recommended in previous studies.^8^^,^^15^^,^^16^ Adapting the approach of the past studies,^6^^,^^7^ an index was derived based on a positive response to the following: (1) not married or cohabitating with a partner; (2) did not live with their children or had nobody to provide emotional or instrumental social support; (3) did not have immediate family members who could provide emotional or instrumental social support; (4) only had face-to-face contact with friends less than once a month or did not have any friends who could provide emotional or instrumental social support; and (5) did not participate in any organizations, religious groups, or committees. A score of zero indicated no social isolation, and a score of 5 indicated individuals who were severely socially isolated. Because the number of participants whose score was 5 points was low, we classified the participants into the following four groups based on their scores: 0, 1, 2, 3, and 4–5 points. Based on previous studies,^7^^,^^13^^,^^17^ we included age (in 5-year bands), age of final educational attainment (≤15 or ≥16 years old), equivalized household income (quintile), activities of daily living (ADL; difficulties in walking, bathing or showering, and using the toilet), comorbidities (total number of medical diagnoses of cancer, heart disease, stroke, hypertension, diabetes, and psychiatric disorders), and the fixed effects of one’s country (England or Japan; Japan served as the reference category) as covariates. ADL was assessed using self-reported limitations in the survey questionnaire with regard to any of the listed activities (ie, walking, bathing or showering, and using the toilet). We dichotomized into “partially dependent (answered “yes” to ≥1)” and “independent (answered “yes” to 0).”

### Analytical approach

First, we conducted a descriptive analysis of participants’ demographic characteristics, health profiles, social isolation, and smoking status. Then, we excluded never smokers (ELSA, *n* = 3,787; JAGES, *n* = 150,050) to examine the association between social isolation and smoking status among ex- and current smokers. Thus, our sample size of the main analysis was 7,092 for ELSA and 68,813 for JAGES, respectively. In our repeated cross-sectional study, a multilevel Poisson regression model with random intercepts was used, with participants at level 1 and the investigation year at level 2. In our model, country difference was treated as the fixed effect and was not treated as a nested effect under the level 2 factors.^18^ After testing for independent main effects between social isolation and smoking status, we added an interaction term between country and social isolation to evaluate the country-based differences in social isolation and smoking status.

In our study, we applied the Markov chain Monte Carlo method based on the Bayesian approach, which enables the calculation of robust estimates when sample sizes within a level 2 unit are small or the response proportion is extreme,^19^ to provide a robust estimate for each parameter with a burn-in of 500 iterations followed by a monitoring chain of 5,000 iterations. Then, we reported the Bayesian 95% credible intervals (CrIs), where the value of interest lies within a 95% probability in the interval, in addition to the parameter estimates. A CrI is a measure of the probability that the true effect estimate would lie within the interval, given the evidence provided by the observed data,^20^ which is different from the conventional confidence interval that indicates the true effect within this interval. We analyzed men and women separately in our study, as the prevalence of smoking in the two countries differed by sex.^21^

Prior to conducting regression analyses, the problem of missing values was addressed using multiple imputations under the missing-at-random assumption. Specifically, missing variables were imputed based on multivariate imputation by chained equations using the following variables: sex, age, educational attainment, equivalized household income, ADL, comorbidities, social isolation, and survey weight (for ELSA only).^13^ The imputation procedure was conducted separately for both countries. After the imputation, we pooled the datasets of the two countries. Rubin’s rules were used to combine the results across the 10 imputed datasets.^22^ We also conducted the same analyses with the complete cases for a sensitivity analysis. Regarding possible intra-correlation from those individuals who participated in both waves, we conducted Poisson regression analyses using only the last observation in the survey (ELSA, 2012–2013; JAGES, 2013). In the ELSA, new study participants were added to maintain the size and representativeness at the 2012–2013 wave. Because we could not identify individuals who participated in both waves in the JAGES study, we used data derived from the 2013 survey wave for participants residing in duplicated municipalities. In this sensitivity analysis, all the variables, including the survey wave, were treated as the fixed effect. The previously mentioned sensitivity analyses were examined using imputed datasets.

The ELSA investigators received ethical approval for all waves of the study from the National Health Service Research Ethics Committees under the National Research and Ethics Service. The JAGES protocols were approved by the ethics committee of Tohoku University (No. 21-40).

## RESULTS

**Figure 1.**
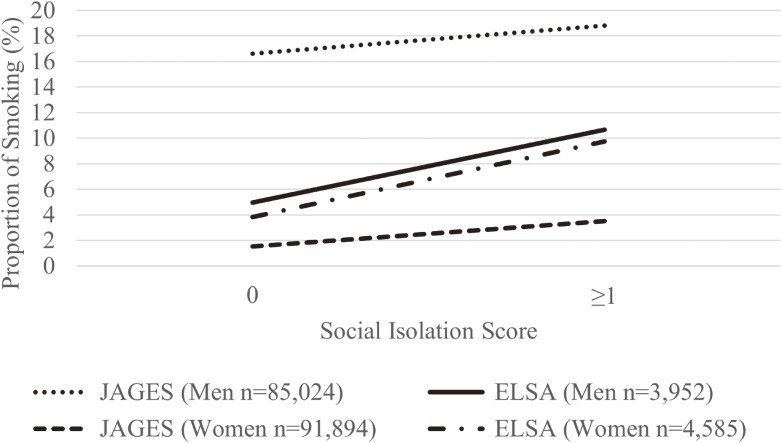
Social isolation and the proportion of current smokers in ELSA and JAGES

Table 1 and Table 2 show demographic characteristics and health profiles of the ELSA and JAGES participants as a function of sex by survey year. Figure 1 also shows men’s and women’s social isolation, respectively, and the proportion of current smokers in the JAGES and ELSA participants. Overall, the proportion of current smokers was higher in men but lower in women in JAGES than in ELSA. In both men and women, more people with social isolation smoked than those who did not, and this was higher in ELSA than in JAGES participants.

**Table 1.** Sociodemographic characteristics of men in ELSA and JAGES by survey year

Men	ELSA				JAGES			
	2010–11		2012–13		2010–12		2013	
	*n*	%	*n*	%	*n*	%	*n*	%
Age, years								
65–69	768	31.4	914	34.9	13,695	29.0	17,262	28.7
70–74	691	28.2	631	24.1	13,781	29.1	17,939	29.8
75–79	499	20.4	544	20.8	10,734	22.7	13,209	22.0
80–84	282	11.5	306	11.7	6,233	13.2	8,088	13.5
≥85	209	8.5	224	8.6	2,846	6.0	3,624	6.0
Age of final educational attainment, years								
≤15	1,034	42.2	1,210	46.2	25,571	54.1	36,252	60.3
≥16	1,297	53.0	1,334	50.9	20,683	43.7	22,767	37.9
Missing	118	4.8	75	2.9	1,035	2.2	1,103	1.8
Equivalized household income, quintile								
1^st^ (highest)	401	16.4	411	15.7	7,833	16.6	9,321	15.5
2^nd^	465	19.0	479	18.3	5,506	11.6	12,331	20.5
3^rd^	498	20.3	534	20.4	11,712	24.8	9,157	15.2
4^th^	526	21.5	588	22.5	7,527	15.9	10,281	17.1
5^th^ (lowest)	330	13.5	328	12.5	8,443	17.9	10,155	16.9
Missing	229	9.4	279	10.7	6,268	13.3	8,877	14.8
ADL								
Independent	2,143	87.5	2,302	87.9	45,240	95.7	55,945	93.1
Partially dependent	305	12.5	317	12.1	984	2.1	1,830	3.0
Missing	1	0.0	—	—	1,065	2.3	2,347	3.9
Comorbidity^a^	0.29 (0.59)		0.25 (0.56)		1.01 (0.78)		0.88 (0.82)	
Smoking status								
Never smoker	597	24.4	664	25.4	11,646	24.6	30,257	50.3
Ex-smoker	1,562	63.8	1,682	64.2	23,416	49.5	18,340	30.5
Current smoker	232	9.5	242	9.2	8,469	17.9	10,632	17.7
Missing	58	2.4	31	1.2	3,758	8.0	893	1.5
Social isolation^b^								
0	759	31.0	834	31.8	9,165	19.4	8,405	14.0
1	655	26.8	683	26.1	9,538	20.2	14,688	24.4
2	341	13.9	363	13.9	11,832	25.0	14,353	23.9
3	112	4.6	140	5.4	6,072	12.8	8,050	13.4
4–5	36	1.5	29	1.1	2,938	6.2	3,519	5.9
Missing	546	22.3	570	21.8	7,744	16.4	11,107	18.5

ADL, activities of daily living; ELSA, English Longitudinal Study of Ageing; JAGES, Japan Gerontological Evaluation Study.

^a^Mean number (standard deviation) of comorbidities. Since the values were rounded off, several percentages do not add up to exactly 100%.

^b^A score of zero indicates no social isolation, and a score of 5 indicates individuals who are severely socially isolated.

**Table 2.** Sociodemographic characteristics of women in ELSA and JAGES by survey year

Women	ELSA				JAGES			
	2010–11		2012–13		2010–12		2013	
	*n*	%	*n*	%	*n*	%	*n*	%
Age, years								
65–69	840	28.7	954	31.1	14,994	27.0	18,673	26.8
70–74	762	26.0	709	23.1	15,947	28.7	20,776	29.8
75–79	581	19.8	663	21.6	12,667	22.8	15,622	22.4
80–84	396	13.5	386	12.6	7,736	13.9	9,594	13.8
≥85	349	11.9	354	11.6	4,236	7.6	4,953	7.1
Age of final educational attainment, years								
≤15	1,255	42.9	1,388	45.3	26,099	47.0	36,905	53.0
≥16	1,554	53.1	1,588	51.8	27,540	49.6	30,829	44.3
Missing	119	4.1	90	2.9	1,941	3.5	1,884	2.7
Equivalized household income, quintile								
1^st^ (highest)	319	10.9	350	11.4	7,251	13.1	8,923	12.8
2^nd^	462	15.8	454	14.8	4,954	8.9	10,852	15.6
3^rd^	608	20.8	629	20.5	10,140	18.2	8,180	11.8
4^th^	690	23.6	784	25.6	7,657	13.8	10,555	15.2
5^th^ (lowest)	702	24.0	681	22.2	11,990	21.6	13,892	20.0
Missing	147	5.0	168	5.5	13,588	24.5	17,216	24.7
ADL								
Independent	2,352	80.3	2,528	82.5	52,505	94.5	64,002	91.9
Partially dependent	574	19.6	534	17.4	1,267	2.3	2,417	3.5
Missing	2	0.1	4	0.1	1,808	3.3	3,199	4.6
Comorbidity^a^	0.26 (0.56)		0.21 (0.52)		0.85 (0.72)		0.72 (0.73)	
Smoking status								
Never smoker	1,232	42.1	1,294	42.2	43,767	78.8	64,380	92.5
Ex-smoker	1,359	46.4	1,496	48.8	2,524	4.5	1,590	2.3
Current smoker	259	8.9	260	8.5	1,622	2.9	2,220	3.2
Missing	78	2.7	16	0.5	7,667	13.8	1,428	2.1
Social isolation^b^								
0	640	21.9	758	24.7	10,978	19.8	10,705	15.4
1	911	31.1	904	29.5	14,943	26.9	19,950	28.7
2	488	16.7	489	16.0	10,922	19.7	15,350	22.1
3	160	5.5	175	5.7	5,067	9.1	6,430	9.2
4–5	32	1.1	28	0.9	1,665	3.0	2,027	2.9
Missing	697	23.8	712	23.2	12,005	21.6	15,156	21.8

ADL, activities of daily living; ELSA, English Longitudinal Study of Ageing; JAGES, Japan Gerontological Evaluation Study.

^a^Mean number (standard deviation) of comorbidities. Since the values were rounded off, several percentages do not add up to exactly 100%.

^b^A score of zero indicates no social isolation, and a score of 5 indicates individuals who are severely socially isolated.

### Smoking (ex- vs current smokers) and social isolation

Results of the sex-specific multilevel Poisson regression analysis for several models are presented in Table 3. Overall, social isolation was significantly associated with current smoking status (reference: ex-smokers); the prevalence ratios (PRs) were 1.06 (95% CrI, 1.05–1.08) for men and 1.08 (95% CrI, 1.04–1.11) for women. In the final model, the interaction term between country (ie, reference: Japan) and social isolation was significant and positive in both men and women; the PRs were 1.32 (95% CrI, 1.14–1.50) for men and 1.30 (95% CrI, 1.11–1.49) for women.

**Table 3.** Smoking status (ex- vs current smokers) and social isolation as a function of sex for the multiply imputed data (multilevel Poisson regression analysis)

Men (ex- vs current smokers)	Model 1			Model 2			Model 3		
	PR	95% CrI		PR	95% CrI		PR	95% CrI	
Social isolation	1.08	1.06	1.10	1.07	1.05	1.09	1.06	1.05	1.08
Country									
Japan				1.00			1.00		
England				0.43	0.35	0.50	0.30	0.22	0.37
Country^*^social isolation (Japan serves as the reference category)							1.32	1.14	1.50

Women (ex- vs current smokers)	Model 1			Model 2			Model 3		
	PR	95% CrI		PR	95% CrI		PR	95% CrI	
Social isolation	1.11	1.08	1.15	1.09	1.05	1.12	1.08	1.04	1.11
Country									
Japan				1.00			1.00		
England				0.41	0.33	0.49	0.28	0.20	0.36
Country^*^social isolation (Japan serves as the reference category)							1.30	1.11	1.49

CrI, credible interval; PR, prevalence ratio.

Model 1, crude model; Model 2, age, educational attainment, equivalized household income, activities of daily living, comorbidity, and country added to Model 1; Model 3, interaction term added to Model 2.

The results of the sensitivity analyses were similar irrespective of the use of complete or multiply imputed data (eTable 1). Also, similar results were observed in the second type of sensitivity analysis (see eTable 2).

## DISCUSSION

To the best of our knowledge, our study is the first to examine country differences in the association between social isolation and smoking. We found that social isolation was more strongly associated with the smoking status of English men than Japanese men. A similar trend was also observed among women.

As expected, the association between a social network and smoking cessation behavior was lower in circumstances where the retail tobacco prices are low. This finding was consistent with a cross-sectional Chinese study reporting that lower levels of contact with friends and relatives were positively associated with current smoking status among women only.^12^ This weak effect of having peers/friends/family members may be attributable to the low tobacco taxes in China (the cost of a pack of 20 cigarettes in China is 1.62 USD).^11^ Therefore, raising tobacco taxes might possibly enhance the network effect of smoking cessation. However, more cross-national comparative studies are required to confirm this possibility.

We also showed that social isolation, defined in terms of marital status (eg, widowed or divorced), poor social networks with friends and relatives, and low levels of participation in social activities, was positively associated with current smoking status in both countries, consistent with previous studies.^12^^,^^23^^–^^25^ Similar to our findings, past studies have shown that being single (which is one indicator of social isolation) is associated with smoking status worldwide.^12^^,^^23^^–^^25^ One longitudinal study conducted across several European countries reported that marital losses (eg, becoming widowed or divorced) were negatively associated with smoking cessation among men and women aged ≥50 years old.^25^ Regarding social networks, a cross-sectional study conducted in China reported that poorer social networks were positively associated with current smoking status among women.^12^ As for social participation, which is another aspect of social isolation, several previous studies have demonstrated it to be positively associated with current smoking status,^12^^,^^26^^–^^28^ which is consistent with our findings.

In recent times, cross-national comparative studies have enhanced our understanding of longevity.^13^^,^^29^^,^^30^ Although Japan is one of the countries with the highest life expectancy rates,^31^ studies that have directly compared the health status of Japanese individuals with those of other countries are scarce. To address this gap in the literature, we examined variables that were directly comparable between countries. To the best of our knowledge, only one study has compared differences in the survival of older adults in Japan and England,^13^ and it showed that smoking status was a stronger contributor to mortality among Japanese men than among English men.^13^ Thus, it is essential to tackle social isolation, as this can improve longevity by mitigating smoking habits.

Our study has implications for public health providers and, thus, for policymakers. Noteworthy differences in England and Japan’s tobacco control policies may account for the differential country-level associations of social isolation with smoking status that emerged in the present study. Indeed, we found that in both sexes, for every 1-point increase in social isolation, English participants were more likely than Japanese participants to be smokers. Moreover, English participants who were less socially isolated were more likely to quit smoking, especially men. Similar results were observed when we performed additional analyses in which we examined the data of never smokers vs current smokers (See eTable 3). These findings can be attributed to higher tobacco taxes and strict smoke-free legislation in England than Japan, which are aspects of tobacco control policies (see eTable 4). First, as previously mentioned, the retail price of tobacco is substantially higher in England than in Japan,^11^ which may, in turn, maximize the social network effect on smoking cessation. Second, the legislation that was introduced in the United Kingdom in 2007 requires all indoor public places to be smoke-free environments.^21^^,^^32^ In contrast, the smoke-free legislation that is in effect in Japan^21^^,^^33^ allows people to smoke in indoor public spaces. Therefore, it is speculated that English smokers who are less socially isolated may be more likely to quit smoking than their Japanese counterparts because of strict tobacco control policies, as shown in eTable 4. In this context, smoking is considered a macro-level norm in countries where tobacco control policies are more lenient. Thus, Japan must enact tobacco-control policies that necessitate an increase in the taxes applicable to tobacco and stricter smoke-free legislation to promote smoking cessation. On the other hand, policies for social isolation also matter. A systematic review suggested that group- or community-based intervention programs are essential for tackling social isolation.^34^ Moreover, another recent systematic review reported that group-based smoking cessation programs were more effective than self-help programs.^35^ In addition to our findings, these studies potentially indicate that interventions for social isolation are effective for smoking cessation among older people. Future studies are expected to substantiate this.

The present study has several limitations. First, there are differences in the designs employed by the two studies (ELSA and JAGES). Specifically, the JAGES respondents were not nationally representative. In fact, the proportion of each smoking status differed between survey waves and, thus, our results might be over- or under-estimated the associations of social isolation with smoking status. In the 2010–2012 survey, 31 municipalities in 12 of the 47 prefectures of Japan were enrolled. In the 2013 survey, 30 municipalities in 14 of the 47 prefectures were enrolled, resulting in 24 municipalities in 10 prefectures participating in both waves. However, the JAGES data came from a nationwide aging study in which more than one-third of the total prefectures (16/47) were enrolled. Additionally, older adults who had a disability were excluded from the JAGES but not the ELSA, which may have led to a potential selection bias. To address the possibility of such a bias, we excluded ELSA participants aged ≤64 years old and controlled for ADL in the regression analysis. Second, there may have been a potential response bias due to the use of self-report questionnaires. For example, the responses to the ADL questions may have been different owing to cultural differences between the two countries. Moreover, the comparability of some of the covariates used in the present study is limited to a certain degree. For example, we measured equivalized household income in terms of quintiles for each country and year of investigation. Thus, participants who had the same percentiles but belonged to different countries could not be compared. However, since the country served as a fixed effect, the possibility of this bias is considered low. Additionally, these variables were also treated as covariates in our regression models. Third, we could not directly compare the effects of tobacco control policies between the two countries because there is no comparative measurement to do so. In Europe, the multidimensional Tobacco Control Scale is widely used to quantify and measure the implementation of tobacco control policies at the country level.^36^ So far, using this scale, several cross-national comparative studies have been conducted to monitor national policy development and implementation.^9^^,^^37^^–^^39^ In Japan, on the other hand, there are no valid scores on the Tobacco Control Scale. Thus, it is expected that future studies will determine which types of tobacco control policies are correlated with the association between social isolation and smoking status using validated scales. Moreover, we could not take into account individual-level factors associated with smoking cessation, such as dependency measures, the amount of tobacco smoked per day, or the number of cigarettes smoked among ex-smokers because these data were lacking in the JAGES survey. Fourth, we could not consider changes in smoking status and social isolation over time because this is a cross-sectional study. It is possible that participants’ smoking status or degree of social isolation changes over time. Besides, we did not assess the duration of smoking cessation among ex-smokers. Thus, future studies are expected to find out this.

In conclusion, we examined the association of social isolation with smoking status in older adults in England and Japan, determining that older people who were less socially isolated were more likely to quit smoking in England than in Japan, possibly explained by the strict tobacco control policies in England. Policies to raise taxes and to enforce smoke-free areas as well as the provision of support for socially isolated individuals are essential to reduce the prevalence of smoking.

## APPENDIX A. SUPPLEMENTARY DATA

eTable 1. Multilevel Poisson regression results on Current smoking (reference = ex-smokers) and social isolation by sex with completed and imputed cases

eTable 2. Smoking status (ex-smokers vs. current smokers) and social isolation as a function of sex for the multiply imputed data (Poisson regression analysis)

eTable 3. Multilevel Poisson regression results on Current smoking (reference = never smokers) and social isolation by sex with completed and imputed cases

eTable 4. Comparison of tobacco control policy between UK and Japan (Tobacco Control Scale; possible range of 0–100. Greater score = stricter)
