# Supplementary material for: Association Between Social Isolation and Smoking in Japan and England
Source: J Epidemiol. 2021 Oct 5;31(10):523–9. doi: 10.2188/jea.JE20200138 (PMC8421201; doi:10.2188/jea.JE20200138)
Supplement: Supplementary file 1 [file je-31-523-s001.pdf]

eTable 1. Multilevel Poisson regression results on Current smoking (reference = ex-smokers) and social isolation by sex with completed and imputed cases

Ex- vs. current smokers	Men						Women					
	Complete			Imputed			Complete			Imputed		
	PR	95% CrI		PR	95% CrI		PR	95% CrI		PR	95% CrI	
Age, years												
65–69	1.00			1.00			1.00			1.00		
70–74	0.84	0.80	0.87	0.84	0.79	0.88	0.91	0.82	1.00	0.93	0.85	1.02
75–79	0.70	0.66	0.74	0.71	0.66	0.75	0.81	0.71	0.91	0.81	0.73	0.90
80–84	0.59	0.55	0.63	0.59	0.55	0.64	0.72	0.60	0.85	0.74	0.65	0.84
≥85	0.49	0.44	0.55	0.50	0.44	0.55	0.50	0.38	0.66	0.63	0.52	0.74
Age of final educational attainment, years												
≤15	1.00			1.00			1.00			1.0		
≥16	1.14	1.09	1.18	1.08	1.03	1.13	1.16	1.06	1.27	1.05	0.97	1.13
Equivalized household income, quintile												
1st (highest)	1.00			1.00			1.00			1.00		
2nd	0.95	0.89	1.01	1.01	0.93	1.09	0.95	0.81	1.11	1.10	0.93	1.27
3rd	0.97	0.92	1.03	1.04	0.96	1.11	0.96	0.81	1.12	1.09	0.94	1.24
4th	1.15	1.08	1.22	1.14	1.06	1.22	1.09	0.94	1.26	1.13	0.98	1.29
5th (lowest)	1.27	1.20	1.35	1.25	1.16	1.34	1.17	1.01	1.35	1.17	1.03	1.32
ADL												
Independent	1.00			1.00			1.00			1.00		
Partially dependent	1.16	1.04	1.30	0.99	0.86	1.11	0.97	0.80	1.17	0.90	0.74	1.07

Comorbidity	0.81	0.79	0.83	0.83	0.81	0.85	0.88	0.82	0.93	0.91	0.87	0.96
Social isolation	1.06	1.04	1.08	1.06	1.05	1.08	1.07	1.02	1.11	1.08	1.04	1.11
Country												
Japan	1.00			1.00			1.00			1.00		
England	0.23	0.19	0.28	0.30	0.22	0.37	0.20	0.16	0.25	0.28	0.20	0.36
Country*social isolation	1.40	1.26	1.55	1.32	1.14	1.50	1.43	1.28	1.59	1.30	1.11	1.49

ADL, activities of daily living; CrI, credible interval; PR, prevalence ratio.

eTable 2. Smoking status (ex-smokers vs. current smokers) and social isolation as a function of sex for the multiply imputed data (Poisson regression analysis)

Ex-smokers vs. current smokers		Men			Women	
Social isolation	1.07	1.03	1.10	1.05	1.004	1.09
Country						
Japan	1.00			1.00		
England	0.30	0.23	0.38	0.24	0.21	0.35
Country*social isolation (Japan serves as the reference category)	1.28	1.10	1.46	1.34	1.17	1.50

The models were adjusted for age, educational attainment, equivalized household income, activities of daily living, comorbidity, and wave fixed effects.

CI, confidence interval; PR, prevalence ratio.

eTable 3. Multilevel Poisson regression results on Current smoking (reference = never smokers) and social isolation by sex with completed and imputed cases

Never vs. current smokers	Men						Women					
	Complete			Imputed			Complete			Imputed		
	PR	95% CrI		PR	95% CrI		PR	95% CrI		PR	95% CrI	
Age, years												
65–69	1.00			1.00			1.00			1.00		
70–74	0.72	0.69	0.75	0.73	0.69	0.76	0.66	0.60	0.73	0.73	0.66	0.79
75–79	0.54	0.51	0.56	0.54	0.51	0.57	0.42	0.37	0.48	0.50	0.45	0.56
80–84	0.48	0.45	0.52	0.48	0.45	0.52	0.31	0.25	0.36	0.39	0.34	0.44
≥85	0.39	0.35	0.44	0.40	0.35	0.44	0.19	0.14	0.25	0.31	0.26	0.36
Education, years												
≤15	1.00			1.00			1.00			1.00		
≥16	1.04	0.998	1.08	1.03	0.98	1.07	1.31	1.20	1.42	1.08	1.00	1.17
Equivalized household income, quintiles												
1st (highest)	1.00			1.00			1.00			1.00		
2nd	0.99	0.93	1.05	1.02	0.95	1.10	0.94	0.79	1.11	1.12	0.95	1.29
3rd	1.03	0.97	1.10	1.07	0.99	1.14	0.99	0.84	1.16	1.13	0.97	1.28
4th	1.15	1.08	1.22	1.12	1.04	1.20	1.44	1.23	1.65	1.24	1.06	1.41
5th (lowest)	1.12	1.05	1.19	1.10	1.02	1.17	1.54	1.33	1.77	1.30	1.13	1.46
ADL												
Independent	1.00			1.00			1.00			1.00		

Partially dependent	1.21	1.08	1.35	0.94	0.83	1.05	1.20	0.98	1.45	1.07	0.88	1.26
Comorbidity	0.89	0.87	0.91	0.92	0.90	0.95	0.87	0.81	0.93	0.99	0.94	1.04
Social isolation	1.05	1.04	1.07	1.11	1.09	1.13	1.53	1.47	1.59	1.37	1.32	1.41
Country												
Japan	1.00			1.00			1.00			1.00		
England	0.44	0.37	0.53	0.78	0.59	0.97	4.68	3.76	5.69	4.20	2.75	5.66
Country*social isolation	1.36	1.22	1.49	1.33	1.16	1.49	1.06	0.95	1.18	1.10	0.94	1.26

ADL, activities of daily living; CrI, credible interval; PR, prevalence ratio.

eTable 4. Comparison of tobacco control policy between UK and Japan (Tobacco Control Scale; possible range of 0–100. Greater score = stricter)

	UK (in 2010)*	Japan (in 2007)**
Price of cigarettes	26	7
Smoke free work and other public places	21	4
Spending on public information campaigns	8	0
Comprehensive bans on advertising and promotion	9	6
Large direct health warning labels	4	4
Treatment to help smokers stop	9	6

* The scores were based on the following webpage:

https://www.tobaccocontrolscale.org/TCS_Graphics/ (Accessed on May.22nd.2020).

** The scores were based on the following report by Dr. Oshima (written in Japanese): https://mhlw-grants.niph.go.jp/niph/search/Download.do?nendo=2006&jigyoId=063031&bunkenNo=200621011A&pdf=200621011A0001.pdf (Accessed on May.22nd.2020). The data is only available for 2005 or 2007.
